# Supplementary material for: Are carbon emissions trading and green financial instruments synergistic? -Comprehensive quantitative research based on content analysis
Source: PLoS One. 2024 Mar 7;19(3):e0298601. doi: 10.1371/journal.pone.0298601 (PMC10919601; doi:10.1371/journal.pone.0298601)
Supplement: S1 Data — (ZIP) [file pone.0298601.s001.zip › Data/地级市Z_F工作报告.zip!/╔╜╢1⁄2/╚╒╒╒╩╨/└┤╫╘╣1⁄2╓┌║┼í╛┬φ┐╦╩2╛▌═°í┐_2014.docx]

各位代表：

现在，我代表市人民政府向大会报告工作，请予审议，并请市政协委员提出意见。

2013年政府工作回顾

刚刚过去的2013年，是经济形势错综复杂、困难和挑战较多的一年。市政府在省委、省政府和市委的坚强领导下，在市人大、市政协的监督支持下，深入贯彻落实党的十八大、十八届三中全会和习近平总书记系列重要讲话精神，把握主题主线，坚持稳中求进、好中求快工作基调，稳增长促转调，打基础利长远，抓改革增活力，办实事惠民生，经济社会实现持续健康发展，一批事关全局和长远的大事取得重要进展。初步预计，全年生产总值突破1500亿元，比上年增长10.6%，三次产业比例调整为8：53.7：38.3。地方公共财政预算收入突破百亿大关，增长15.5%。港口吞吐量突破3亿吨，相当于三年再造一个亿吨大港。进出口总值突破300亿美元，总量、增幅分列全省第3位和第2位。固定资产投资增长20%、规模以上工业增加值增长11.5%、社会消费品零售总额增长13%，城镇居民人均可支配收入和农民人均纯收入分别增长10%和11%。城镇登记失业率2.3%，居民消费价格涨幅2.2%。

一、经济发展稳中有进、稳中向好。一是着力强化农业基础地位。立足粮食安全和农产品有效供给，积极开展高产创建活动，深入推进农业结构调整，大力发展现代农业。粮食产量连续9年获得100万吨以上好收成。新增特色种植业面积10.6万亩，国家级农业标准化示范区达到10个，东港区成为“全国特色农业知名品牌创建示范区”。新发展规模化养殖场81家。农业生产综合机械化水平达到84%。大力培育新型农业经营主体，农民专业合作社、家庭农场分别发展到2844家和99家，新增销售收入过亿元龙头企业15家。投资13亿元建成各类水利工程1954处，新增蓄水能力1200万立方米。植树造林13.7万亩，创建为“山东省森林城市”。加大海洋资源保护修复力度，入选首批国家级海洋生态文明示范区。全面落实强农惠农政策，发放各类惠农补贴5.58亿元。二是积极推动工业结构优化升级。加快传统工业升级改造，大力发展新兴产业，着力培育优质产业集群。全市主营业务收入过百亿的企业发展到5家，其中年内新增2家，过50亿元的达到11家。装备制造业、高新技术产业占比分别提高1.8和1个百分点，高耗能行业占比下降0.8个百分点。现代派沃泰自动变速箱二工厂、威亚发动机增产、万方板业镀锌彩涂板等项目建成投产，新增汽车变速箱产能50万台、发动机10万台，汽车及零部件制造业产值超越钢铁，成为全市第一大产业。完善以企业为主体的科技创新体系，设立了1000万元的引智专项资金，新建市级以上企业技术中心19家、工程技术研究中心16家、海外人才工作站3处、引智技术转移中心3处，日照港集团博士后科研工作站获准设立。三是加快发展服务业。完成服务业投资630亿元，服务业增加值比重提高0.5个百分点。凌云工贸冷链物流配送中心、凯达农村物流分拨中心等项目建成投运，物流业增加值、投资占比居全省前列。全年旅游人数达到3153万人次，实现旅游总收入210亿元，分别增长11.6%和14.6%。电子商务、文化创意等新兴业态破题起步，日照国际铁矿石交易中心、新商所蓝海商品交易中心投入运营，窝窝商城总部落户日照，文化创意与服务外包产业园项目启动。四是加大金融财政支撑力度。华夏、浦发银行日照分行开业运营，日照银行在临沂、潍坊设立分行，新引进保险、期货、证券机构6家，新增小额贷款公司6家，全市信用总量和贷款余额分别增加690亿元和190亿元。加大对小微企业扶持力度，设立了中小企业公共服务中心，拿出2.5亿元财政间歇资金作为信用保证金，撬动银行向中小微企业发放“财政增信优惠贷”9.7亿元。扎实做好“营改增”试点工作，全面落实小微企业免征营业税和增值税政策，取消和停征部分行政事业性收费，共减轻企业税费负担4亿多元。五是强化环保、土地等要素约束。启动实施了总投资37.9亿元的10项环保重点治理工程，治理目标向社会公开承诺，五莲县、东港区1226家园外石材企业全部关停；水泥立窑生产线全部淘汰；电力、水泥行业脱硝基本完成；3家污水处理厂投入试运行。强化节约集约用地，加大闲置低效土地处置力度，共收储土地141宗、7653亩。

二、大项目推进取得突破。围绕实施“双十•一城”工程，开工建设大项目149个，完成投资470.3亿元。日照机场获国务院、中央军委批复，6个土石方试验段和2个爆破工程标段开工。日照钢铁精品基地一期工程开工建设，部分拆迁安置楼主体工程竣工。石臼港区规划调整论证研究工作获交通运输部同意，南区泊位堆场等工程正在推进。完成港口投资39.6亿元，新建启用6个泊位，日照、岚山口岸通过世卫组织核心能力达标验收，并创建为“全国运行管理先进口岸”。山西中南部铁路已完成境内投资计划的85.5%。青日连铁路完成环评审批和风险评估。日照至济南城际铁路列入全省城际轨道交通网补充规划。国际海洋城已完成基础设施投资16.6亿元。204国道开发区至汾水段、日兰高速龙山互通立交、336省道莒县至沂水段改建工程竣工通车，山海路西延完成工程量的95%，613省道西延一期工程全面开工。巨峰水库向岚山供水工程投入使用，龙潭沟水库主体工程基本完成，“沭水东调”和沭河治理工程开工建设。220千伏石井、110千伏荻水等7项输变电工程建成投运。

三、城乡一体化发展提速。一是城市建设初步实现了由外延扩张向内涵提升的转变。加快完善中心城区市政设施，完成城建重点工程投资88.1亿元，中心商务区安置楼主体基本完工，淄博路道路及地下设施、尚城花园安置区、招银大厦开工建设。对石臼老城区11条道路进行了设施改造和绿化提升，新建改造城市道路7条，新增城市绿化面积120万平方米，海岸带整治取得阶段性成果。商品房开工面积692万平方米，增长13%。调整完善城中村改造政策，完成拆迁面积65万平方米。加大城管执法力度，强化扬尘整治和建筑垃圾运输管理，市容环境得到改善。二是提升城镇化水平。深入推进户籍管理制度改革，新落户城镇人口21710人、比上年增加5171人，城镇化率达到51.34%、提高1.5个百分点。市县财政投入6000万元对6个省、市级示范镇基础设施进行补助。全面推进城乡环卫一体化，建成区以外村庄全部配备环卫设施和保洁人员，新增国家级生态乡镇10个。新建改建农村公路750公里，莒县、五莲农村公路网化示范县工程通过省验收。建成电气化镇5个、电气化村381个。农村自来水普及率提高到95%。

四、改革开放迈出新步伐。一是深入推进重点领域改革。下大力气改善营商环境，取消、下放行政审批事项113项，出台了工商登记改革18条措施，全市私营企业户数增长15.6%，注册资本增长35.2%，分列全省第5位和第3位。食品药品、工商、质检管理体制改革启动实施，公共资源交易实现集中统一规范管理。农村产权制度改革试点完成，面上工作全面启动。水利工程确权划界基本完成。积极推动投资主体多元化，日照常春藤股权投资基金成为我市首支、全省最大的私募股权基金，组建了青苹果置业、兴城置业、东城置业等混合所有制企业。大力推进企业上市、挂牌和发债融资，实现直接融资55亿元，是上年的1.8倍；新增场外市场挂牌托管企业25家，其中农业龙头企业10家。农村信用社改革破题，五莲农商行挂牌成立，莒县农商行获银监会批准。二是全面扩大开放合作。努力营造“安心、开心、舒心、放心”的创业投资环境，外向型经济逆势增长，再创新高。实际到账外资5.26亿美元、增长25%。实现境外投资1.32亿美元、增长44%。成功举办了“韩国周”，赴港台日韩等地开展招商推介活动，新引进企业249家，注册资本78.6亿元。首批对口援建新疆麦盖提县三年任务圆满完成。

五、保障和改善民生取得新成效。各级财政用于改善民生的支出达到94.96亿元，占财政支出的60%，12件为民办实事重点项目全面完成。全市新增城镇就业5.78万人、农村劳动力转移就业3.7万人。在全省率先开展被征地农民参加城镇职工基本养老保险。为2413名乡村医生办理了社会养老保险。开展了新农合大病保险试点，城镇基本医疗保险实现省内异地就医网上即时结算。全面提高了企业退休人员基本养老金、失业保险金、最低工资标准和城乡低保、“五保”供养、孤残儿童救助、城乡居民医保财政补助标准。加快社会养老服务体系建设，新增养老床位2726张。对5433户困难家庭建立了各级干部直接联系帮扶制度。新改扩建居民小区全面配备公共服务设施。新购置新能源公交车100辆，新建公交候车亭125个。新设改建便利消费网点74处。开工建设保障性住房11320套，完成年度任务的113%。投入14亿元，新建改建学校、幼儿园231所，新增校舍面积、高考本科录取人数、职业院校技能大赛成绩创历史新高。深入开展“文化建设年”和全国文明城市创建活动。承办“十艺节”工作荣获“组织奖”，“千百重点文化工程”获首届山东省文化创新奖，市图书馆主体工程竣工。群众体育活动蓬勃开展，激光雷迪尔级帆船世界锦标赛精彩成功。全面完成了人口责任目标，人口自然增长率控制在3.28‰。市人民医院通过全国三甲综合医院评审。在五莲县开展了县级公立医院综合改革试点。深化平安日照建设，不断完善警务“大回访”机制，扎实做好信访工作，我市再次荣获“全国社会管理综合治理优秀市”。深入开展安全生产大检查和食品药品安全专项整治，完善应急管理机制，安全生产形势稳定好转。积极推动全国农村社区管理和服务创新实验区工作，建成农村社区350个、综合服务中心128处。国防动员、双拥共建和民兵预备役建设不断深化。市人防应急指挥中心建成启用。《日照市志》二轮修编启动。妇女儿童、红十字、民族宗教、打击走私、司法仲裁、国家安全、外事侨务、档案、气象、水文等工作取得新成绩。

六、政府自身建设不断加强。扎实做好党的群众路线教育实践活动前期工作，深入排查整治“四风”问题，大力开展“庸懒散”专项治理，保持了求真务实、真抓实干的良好作风。积极完善政府决策机制，对城市建成区内土地使用、机关事业单位人员招聘等重要公共资源配置和社会关注的问题，一律由市政府常务会议集体审定。自觉接受人大法律监督和政协民主监督，认真做好人大代表建议、政协提案办理工作，办复率和满意、基本满意率均达到100%。成立了市政府行政复议委员会，对47个部门、单位的行政复议权进行集中。扎实做好市长公开电话、行风在线、网络问政等工作，保持了民意诉求渠道畅通。严格落实党风廉政建设责任制，扎实推进惩防体系建设，加大行政监察、审计监督力度，从严查处违纪违法案件。全面落实中央八项规定，严查公款吃喝、公车私用等违规行为，“三公”经费明显下降。

各位代表！

过去一年我市经济社会发展成绩来之不易。这是省委、省政府和市委坚强领导的结果，是市人大、市政协和社会各界监督支持的结果，是全市干部群众同心同德、狠抓落实的结果。在此，我代表市人民政府，向辛勤工作在各条战线上的全市人民，向各级人大代表、政协委员，向各民主党派、工商联、无党派人士、各人民团体，向离退休老领导、老同志，向驻日照人民解放军和武警官兵，向所有关心支持日照建设与发展的社会各界人士和海内外朋友，表示衷心的感谢并致以崇高的敬意！

在肯定成绩的同时，我们也清醒地认识到面临的挑战和问题，主要是：产业发展不充分、经济总量小、科技创新能力弱、城乡发展不平衡；制约发展的体制机制问题还比较突出；教育、医疗、就业、社会保障、环境保护等工作有待进一步强化；政府服务水平和行政效能需要进一步提高。对此，我们一定高度重视，采取更加有效的措施，努力加以解决。

2014年政府工作任务

今年是贯彻落实党的十八届三中全会精神、全面深化改革的第一年，也是完成“十二五”规划的关键一年。全市经济社会发展的总体要求是：深入贯彻党的十八大、十八届三中全会和习近平总书记系列重要讲话精神，坚持稳中求进、改革创新工作基调，着力用好“蓝红金”国家战略机遇，着力稳增长、抓改革、调结构、强蓝区、优生态、惠民生、保稳定，着力推动经济社会率先创新优质发展，为实现“一个率先、五个日照”奋斗目标打下坚实基础。

主要预期目标是：生产总值增长10.5%左右，公共财政预算收入增长10%左右，固定资产投资增长20%左右，社会消费品零售总额增长13%左右，进出口总值增长6%左右，规模以上工业增加值增长12.5%左右，城镇居民人均可支配收入、农民人均纯收入均增长10.5%左右。城镇登记失业率控制在3%以内，居民消费价格涨幅控制在3.5%左右，全面完成年度节能减排约束性目标。

一、推进全面深化改革。按照正确、准确、协调、有序的总要求，认真落实市委学习贯彻党的十八届三中全会精神、全面深化改革的意见，稳扎稳打推进改革。一是扎实推进行政管理体制改革。加大简政放权力度，改革工商登记制度，推进行业协会、中介组织与行政机关脱钩，抓好涉企收费清理整顿，努力让全社会感受到市场环境、创业条件在一天天好转。完成市县政府机构改革任务，抓好扩权强镇试点，基本完成生产经营类事业单位转为企业或社会组织改革。加大政府向社会组织购买服务力度，凡是适合市场化提供、社会能够承担的公共服务，都要引入竞争机制，向社会购买；凡是花钱直接能办的事情，不能增编养人去办。二是着力激发和释放各类市场主体活力。坚持权利平等、机会平等、规则平等，在市场准入、融资服务等方面对各种所有制企业实行同等待遇。加大力度发展民营企业，积极推动民间资本进入基础设施、基础产业、金融服务和教育、医疗、养老、健康等领域。大力发展混合所有制经济，鼓励国有资本、集体资本、非公有资本交叉持股、融合发展。三是深化投融资体制改革。充分利用多层次资本市场拓展直接融资渠道，鼓励企业在境内外上市，支持上市企业再融资，增加债券融资的种类和数额。设立日照大宗商品交易中心，提升港口集散功能。以合作社为平台探索开展资金互助业务，拓展农民贷款渠道。大力开展“信用日照”建设，打造各行业信用平台，构建诚信服务体系。积极引进金融机构来我市落户，推进日照银行跨区域发展，完成农村信用社银行化改革。四是加快农村综合改革。扎实推进农村土地承包经营权、农民宅基地使用权、房屋所有权确权登记颁证和集体经济组织股份制改革、小型水利工程产权制度改革，赋予农民更多的财产权利。健全农村土地流转制度，鼓励土地承包经营权向专业大户、家庭农场、农民合作社、农业企业有序流转。深化供销社改革，实施“新型农业社会化服务平台创建行动”。五是推进财税制度改革。完善全口径政府预算体系，推动预算公开透明，开展“三公经费”预决算公开试点。推进国库集中支付改革，扩大区县直接支付范围，全面推开乡镇集中支付。做好“营改增”扩围和消费税、房产税、资源税改革准备工作。大力调整财政支出结构，完善转移支付资金使用办法，提高使用绩效。

二、坚决打好转方式调结构这场硬仗。一是坚持“三个导向”，加快发展现代农业。始终把三农工作摆在重中之重的位置，以解决好地怎么种为导向，积极培育专业大户、家庭农场、农民合作社和龙头企业，加快构建新型农业经营体系。以缓解地少水缺的资源环境约束为导向，加强农业科技创新，提高农业规模化、标准化、产业化、机械化和品牌化水平。深入开展高产创建活动，确保粮食总产稳定在100万吨以上。大力发展茶叶、林果、瓜菜、桑蚕、黄烟等优质特色农业，新增高效种植业面积3万亩。积极推动传统渔业转型升级，加快发展现代渔业。抓好六大现代畜牧产业示范区建设，创建现代畜牧业示范市。以满足吃得好吃得安全为导向，大力发展优质安全农产品。重点抓好国家级农业标准化示范项目、省级现代农业示范区、无规定动物疫病区、农产品质量安全示范县和生态农业与农村新能源示范县建设，加强农产品质量安全监管，努力让老百姓吃上安全放心的农产品。二是以“产业链延伸”为抓手，推动优势产业向优质产业转变。坚持把日照钢铁精品基地建设作为“头号工程”，年内力争实现投资85亿元；扎实做好配合服务工作，加快拆迁安置区建设。抓好万方板业镀锡板等钢铁深加工和新材料项目建设，推动钢铁产业向高端高质高效跨越。抓好威亚发动机四工厂、派沃泰变速箱三期、五征60万套农业机械等重点项目建设，做大做强汽车零部件制造业集群。推进森博30万吨高档液体包装纸板、百亮集团260万条高性能轮胎、华泰精细化工、海汇环保装备等项目，加快传统工业转型升级。三是以现代服务业为重点，推动服务业提速提效。支持企业剥离非主营业务，加快培育电子商务、现代物流、创意设计、信息软件、服务外包等新兴业态，力争服务业比重提高1个百分点以上。开工建设文化创意与服务外包产业园，抓好窝窝商城互联网产业基地等重点项目。全面落实商贸流通企业用电、用气、用水、用热与工业同价政策，积极培育各类专业市场，新建4处公益性农产品批发市场，推进农超对接和“生鲜配送进社区”工程。抓好尚城花园、灯塔文化广场、九仙山南麓片区等特色旅游项目建设，实施乡村旅游业户“改厕改厨”工程，提升乡村旅游层次。大力推进信息化在各领域的应用，加快打造“智慧日照”。四是积极实施创新驱动发展战略。坚持引资与引智、引技相结合，鼓励企业与国内外各类院校、科研机构加强合作，大力引进领军人才与创新团队。强化企业在技术创新中的主体地位，健全产学研协调创新机制，加快企业技术中心、工程技术研究中心和创新联盟等平台建设，力争高新技术产业产值增长18%以上。五是努力塑造开放型经济发展新优势。加大对重点产业、重点区域、重点客商的招商力度，加强与现代、金鹰、华润、中石油、中石化、中远等大企业的战略合作，立足现有企业、商会和行业协会扩大以商招商，加快引进高端制造、高新技术、战略性新兴产业及港口等基础设施项目。继续做好综合保税区争取工作，完善各类园区配套设施，创新管理体制机制，打造一流开放载体。积极参与中日韩地方经济合作示范区、自贸区和丝绸之路经济带、海上丝绸之路建设。深入实施以质取胜和市场多元化战略，优化出口商品结构，稳定和扩大国际市场份额。鼓励企业“走出去”，推动本土企业国际化。

三、锲而不舍地抓好大项目。对事关全局和长远的大事，坚持一年接着一年干，一件一件抓落实。（1）抓好日照机场飞行区、航站区、综合服务区建设，开工机场高速，推进奎山客运枢纽等配套工程，启动山东西路客运枢纽项目。（2）加快石臼港区规划调整，力争用四年时间，完成市区铁路货运南移和港口布局调整工作，实现铁路货运“南进南出”、货物吞吐“北集南散”、布局优化与装备、管理提升一步到位。抓好30万吨级原油码头扩建、30万吨级矿石码头等8项港口重点工程建设，完成投资35亿元。（3）完成山西中南部铁路、613省道西延一期、山海路西延、日兰高速路面大修和日照收费站扩容改造；开工青日连铁路、潍日高速日照段和222省道西湖至岚山段改建工程，力争开工岚临高速、潮石路拓宽改造项目，争取启动613省道西延二期工程；积极推动日照—临沂—曲阜城际铁路建设。（4）加快实施“沭水东调”工程，争取2015年全线贯通。建成“北水南调”三期工程，新增日供水能力20万立方米。（5）新建续建220千伏西峪等21项电力基建重点工程，优化提升电力保障能力。

四、积极稳妥推进新型城镇化。坚持以人的城镇化为核心，加大城乡统筹力度，加快人口向城镇集聚、基础设施和公共服务向乡村延伸。一是围绕完善功能、提升内涵，抓好城建重点工程建设。加快中心商务区建设，完成淄博路地下联络通道工程和人防工程主体，尽快形成城市街区规模。建成启用市图书馆，完成丁肇中科技馆主体工程。推进城中村连片改造，抓好青岛路、北京路、菏泽路等6个片区改造。抓好山海路至森林公园段海洋岸线生态恢复。完成泰安路、东营路等6条城市道路和中心商务区10条区间道路改造建设任务。加快公交系统升级改造，新购置150辆新能源公交车，建设3处公交综合停车场，新建100个候车亭，启动智能公交系统建设。二是统筹生产、生活、生态空间布局，提升城镇建设管理水平。按照生产空间集约高效、生活空间宜居适度、生态空间山清水秀的总要求，做好城镇化发展规划编制工作，进一步优化城镇布局和形态。抓好全国发展改革试点镇和省、市级示范镇建设，落实财政补贴资金，提升基础设施和公共服务配套水平。切实加强建筑质量管理，努力打造百年建筑。深化户籍制度改革，完善城市社区功能，积极稳妥地推动人口向城镇集聚。培育各具特色的产业体系，增强城镇产业支撑能力，拓展农民进城就业空间。理顺城管执法体制，提高执法服务水平，加大市容环境、建筑噪声、垃圾运输、交通秩序管理力度，为市民营造安宁安全的生活环境。三是继续改善农业农村基础设施。完成龙潭沟水库建设，实施3座中型水库和49座小型水库除险加固工程，新建600处小微型水源和13处重点河道治理及拦蓄水工程，实施4个小农水重点县、2个水土保持治理试点县工程，扩大改善灌溉面积31万亩，建成高标准农田25万亩。新建扩建农村自来水工程24处，打机井100眼，解决12万农村居民的饮水困难和饮水安全问题。新建改建县乡村道路300公里、高标准电气化村301个、农村社区综合服务中心12处，进一步改善农村生产生活条件。

五、深入推进“生态日照”建设。扎实开展“生态建设年”活动，崇尚自然质朴，建设自然生态宜居城市。一是坚持用制度保护生态环境。严格落实主体功能区制度，强化对水源、山体、森林、湿地、海洋和岸线的强制性保护，在重要生态功能区、敏感区划定生态红线，慎砍树、不填湖，让尊重自然、顺应自然、保护自然的理念落地生根。强化安全、环保、土地硬约束，实施污染物排放总量控制，对新上项目实行等量或减量替代；严格土地市场管理，盘活存量闲置土地，鼓励建设多层标准厂房，提高单位土地投入强度；实施最严格的水资源管理制度，推进日钢海水淡化项目，加大中水回用，建设节水型社会。二是集中整治重点企业、重点区域。深入推进饮用水源地上游环境综合整治，加大破损山体、废弃矿山生态修复力度，对进园石材企业全部配套治污设施；全面落实污染排放重点企业综合治理措施，确保10项环保重点工程完成投资20亿元，实现减排烟尘9700吨、二氧化硫2000吨、氮氧化物5400吨、化学需氧量8650吨、氨氮948吨的预期目标。三是加大节能减排力度。对电力、钢铁、石化、造纸等重点行业加快淘汰落后产能，继续推进建筑节能改造。建成市餐厨垃圾处理厂和莒县、五莲、海洋城4处污水处理厂，启动垃圾焚烧发电、河山污水处理项目建设，加快污水管网配套完善。抓好工业废气、机动车尾气、城市扬尘整治，加强对重点河流断面的污染防治与监管，完成崮河、淤泥河人工湿地建设。四是加强生态宜居环境建设。实施城市林荫工程和裸露土地整治工程，对市区28条道路、37处公园绿地、54个庭院小区、28处停车场进行绿化提升，整治裸露土地220万平方米，新建一批街头公园。加快荒山、水系和路网绿化，积极创建“国家森林城市”。深入开展生态文明区县、水生态文明城市和生态文明乡村创建活动，建立城乡环卫一体化运转长效机制，完成8个镇、27个新型农村社区的污水处理设施建设，改善乡村人居环境。

六、切实加大保障改善民生力度。坚持把“让老百姓过上好日子”作为一切工作的出发点和落脚点，多做雪中送炭的工作，努力让老百姓感受到日子越过越好，让困难群众生活有盼头。

一是完善就业创业体系。（1）突出抓好以高校毕业生为重点的青年就业和农村转移劳动力、城镇就业困难人员就业，加大基层公共管理和社会服务岗位开发力度，新增城镇就业2.65万人，农村劳动力转移就业2万人。（2）设立4000万元扶持资金，扶持创业带动就业1.75万人。（3）提高最低工资标准，构建和谐劳动关系。

二是强化“兜底”保障。（1）继续做好被征地农民参加城镇职工养老保险工作。（2）整合城乡居民基本养老、医疗保险，提高财政补助标准，提升大病保障水平，加快构建城乡一体的社会保障制度。（3）建好管好用好保障性住房。开工建设保障性住房12300套，改造农村危房2000户，发放租赁住房补贴2500户；改进住房公积金提取、使用、监管机制，新增缴存职工9000人，发放贷款4亿元。（4）健全社会养老服务体系。完成2处区县福利中心建设，改造提升乡镇敬老院16处，建设农村幸福院100处，推动公建民营、民办公助等养老方式迈出更大步伐。（5）提高城乡低保和农村五保供养水平，做好困难家庭联系帮扶工作。（6）实施助残安居工程。扶持400个残疾人家庭创业增收，帮助300户贫困残疾人家庭改造危房，为8300名低保重度残疾人发放生活补贴，为500名贫困残疾人免费提供辅助器具，对190名残障儿童进行康复救助。（7）实施库区移民后期扶持项目179个，抓好青峰岭水库移民避险解困工程。

三是优先发展教育事业。（1）深入实施“扩改提”工程，扩建提升城区学校10所。（2）启动第二轮学前教育三年行动计划，新建改建城乡幼儿园50处。（3）坚持“三线推进”义务教育均衡发展，完成农村义务教育学校标准化建设投资6.8亿元。（4）推动普通高中学校全部创建省、市级规范化学校。（5）抓好职业学院、电大、水产技校三校合并，创建2所国家级中等职业教育改革发展示范学校。（6）加快教育信息化步伐，建设50个数字校园。

四是提升医疗卫生保障水平。（1）深化医药卫生体制改革，逐步将非政府办基层医疗卫生机构纳入基本药物制度范围，力争全面启动县级公立医院综合改革。（2）建立乡村医生养老保障和准入退出制度，乡村医生养老保险覆盖率提高到70%以上。（3）实施“健康快车”行动，对常见病、多发病、慢性病进行筛查。

五是全面发展社会事业。扎实做好全国文明城市创建工作，深入开展“德润港城、善行日照”主题道德实践活动，提高市民思想道德素质和城市文明水平。完善提升100处镇村文化设施，在600个行政村配备体育健身设施。7所市属和驻日照高校的文体设施免费向社会开放。加大文化遗产保护力度。办好2014中国“航海日”活动和第23届省运会帆船帆板比赛。推进文化市场综合执法，净化社会文化环境。认真落实人口目标管理责任制和计划生育利益导向政策，提高出生人口素质。做好国防动员、双拥共建、民兵预备役建设和人防工作，落实优抚安置政策，促进军民融合深度发展。抓好《日照市志》二轮修编。积极发展慈善、防震减灾等事业。

六是推动社会治理创新。深入推进平安日照建设，完善全方位、全天候、全员化的维稳体系，强化社会治安综合治理。做好新形势下的信访工作，把解决涉法涉诉信访问题纳入法治轨道。完善普法教育和社区矫正机制，加强法律援助，推广仲裁制度，提高法治日照建设水平。建立价格调节基金制度，增强政府调控能力。抓好物业管理改革，建设宜居小区。推进农村社区管理和服务创新实验区建设，依法管理宗教事务，严厉打击走私贩私。做好第三次经济普查工作。严格落实安全生产责任制，完善应急管理机制，强化食品药品安全监管，保持社会和谐稳定。

七、大力加强政府作风建设。坚持“打铁先得自身硬”，以踏石留印、抓铁有痕的劲头抓好作风建设。

一是在转作风上下功夫。深入学习习近平总书记系列重要讲话精神，扎实开展党的群众路线教育实践活动，坚决扫除作风之弊、行为之垢，形成及时发现和解决作风问题的长效机制。加强调查研究，接地气、连民心，察实情、办实事，始终保持同人民群众的血肉联系。大力弘扬求真务实精神，坚持从一件件具体事情抓起，一步一个脚印，铺下身子抓落实。牢固树立正确的政绩观，多做前人栽树、后人乘凉的工作，创造经得起实践、历史和人民检验的业绩。

二是在依法行政上下功夫。严格按照法定权限和程序行使权力、履行职责。认真落实公众参与、专家论证、社会风险评估等制度，不断提高行政决策的科学化、民主化水平。健全行政执法责任制，强化执法监督，提高公正执法、文明执法水平。完善政府信息公开制度，保障公民知情权、参与权、表达权和监督权，让权力在阳光下运行。自觉接受人大法律监督和政协民主监督，重视司法、舆论和社会公众监督，及时听取各民主党派、工商联、无党派人士、各人民团体和人民群众的意见。

三是在提高行政效能上下功夫。进一步转变政府职能，强化政府公共服务、市场监管、社会管理和环境保护职责，加快法治型和服务型政府建设。大力弘扬尽心尽责、创新创优的工作精神，雷厉风行的工作作风，敢于担当的工作锐气，提高政府执行力、落实力。推进政务服务体系建设，努力为企业和群众提供优质高效便捷服务。

四是在反腐倡廉上下功夫。扎实推进惩防体系建设，支持监察、审计部门依法行使监督权，完善权力运行制约和监督体系，深入开展反腐倡廉教育，从严查处违纪违法案件。政府领导班子成员要带头遵守廉政准则，认真履行党风廉政建设责任制，克己奉公，廉洁自律，带好队伍。坚持政府带头过紧日子，狠刹奢靡之风，从严控制公用经费支出，确保财政供养人员和“三公”经费只减不增，以实际行动取信于民。

各位代表！

新的一年，改革发展任务十分艰巨。让我们紧密团结在以习近平同志为总书记的党中央周围，高举中国特色社会主义伟大旗帜，在中共日照市委的坚强领导下，凝聚共识，汇聚力量，扎实苦干，不断开创经济社会改革发展新局面，为谱写全市人民美好生活新篇章而努力奋斗！
